# Workplace stressors and burnout among healthcare professionals: Insights from the pandemic and implications for future public health crises

**DOI:** 10.1038/s41598-025-21540-2

**Published:** 2025-11-26

**Authors:** Elahe Daneshvar, Steffen Otterbach

**Affiliations:** Institute for Healthcare and Public Management (530), University of Hohenheim, Stuttgart, Germany

**Keywords:** Healthcare professionals, Burnout, Mental health, Workplace stressors, COVID-19, Health care, Health occupations, Risk factors

## Abstract

This study evaluates the prevalence of burnout among healthcare professionals (HCPs) during the COVID-19 pandemic in Iran and examines its association with key occupational stressors (workload, job control, and leadership communication). Furthermore, it proposes relevant organisational interventions for future pandemic preparedness. A cross-sectional survey of HCPs (N = 723) was conducted in four hospitals in Tehran during the third peak of the COVID-19 pandemic in Iran, including the Copenhagen Burnout Inventory and items on perceived workload, job control, and leadership communication. The study found that 67.41% of HCPs reported substantial symptoms of burnout (moderate-to-high burnout, i.e. CBI ≥ 50), with a mean score of 59.6 points. The prevalence of burnout (CBI ≥ 50) was prominent across all three dimensions—personal, work, and patient-related—at 72.86%, 69.87%, and 65.37%, respectively. The analysis demonstrated significant associations between burnout and the three foundational workplace stressors. Multivariate linear regression analysis showed that frontline, female, and married HCPs reported the highest levels of burnout. This study provides practical implications for healthcare organisations and policy makers, highlighting the need for targeted organizational interventions that could mitigate burnout during ongoing and future health crises.

## Introduction

Healthcare Professionals (HCPs) are particularly susceptible to health distress and burnout due to their work environment, which is often fraught with stressors such as time pressure, heavy workload, limited job control, and lack of social and leadership support^1–4^. The World Health Organisation (WHO), recognizing the severity of the issue, has classified burnout as an occupational phenomenon in the most recent International Classification of Diseases (ICD-11), emphasizing its roots in chronic workplace stress that should not be applied to experiences in other domains of life.

Among the occupational factors contributing to burnout, high job demand, low job control, and low job support are known to erode staff well-being and result in adverse health outcomes. Job demand mainly refers to long working hours and increased workload; job control is the amount of the latitude that workers have over their tasks and conduct^5^. During the pandemic, the sudden and unexpected surge in the number of COVID patients led to an abrupt transformation of HCPs’ work environment, a change that decreased HCPs’ perception of control over the conditions of their work. The mismatch between excessive workload and limited job control puts HCPs in a state of anxiety, leading to the physical and mental exhaustion that are at the core of burnout^6^. However, leadership and peer support can play a major role in alleviating or exacerbating burnout among HCPs, particularly in high-pressure situations like the pandemic^7,8^. By the same token, management practices that hinder effective communication within the organization can create a sense of not being heard or supported, increasing work stress and the risk of burnout^4^.

The COVID-19 pandemic has intensified workplace stressors in healthcare systems, where burnout was already pervasive^3,9,10^. Addressing HCPs’ burnout is therefore crucial, given its wide-ranging and dire consequences for health system. Burnout not only compromises patient safety—leading to a higher incidence of medical errors and diminished quality of care— but also takes a severe toll on HCPs’ themselves, increasing the risk of depression, substance abuse, and even suicide. Moreover, its consequences extend to healthcare institutions, resulting in substantial financial burdens, reduced productivity, and high workforce turnover, including early retirements.^11,12^.

Recent studies have consistently documented high rates of severe burnout among HCPs worldwide^13^. For instance, a comprehensive study conducted in 85 countries revealed that 51% of HCPs treating COVID-19 patients experienced severe burnout^9^. In the United States, a study assessing the mental well-being of intensive care unit (ICU) providers across all 50 states found a burnout rate of 58%. This study calls attention to the significant association between burnout and poor communication with supervisors^14^. Furthermore, other U.S.-based studies identified a link between burnout in radiation oncologists and a lack of control over workload during the pandemic^15^. Studies conducted in Spain and Germany during the same period found that job strain, particularly heavy workload and extended working hours, contributed significantly to burnout^16,17^. Studies from Italy, Romania, China, Korea, and India, have painted a similar picture of burnout in healthcare settings with alarmingly high levels of burnout^18–22^.

While these studies underscore the extensive research on the association between HCPs’ burnout and the COVID-19 pandemic, gaps remain in integrating foundational work stressors into the study of burnout during the pandemic. Additionally, data on factors associated with burnout are often inconsistent, particularly in middle- and low-income economies. Iran faced significant HCPs’ losses due to COVID-19, ranking 12th and 8th worldwide in related deaths^23^ . In Tehran, which accounted for 50% of the country’s infections, HCPs represented 17% of those affected^24^, underscoring the urgency of addressing burnout in this critical workforce.

This study aims to address these gaps by examining how fundamental work stressors contribute to HCPs’ burnout within the context of a resource-constrained healthcare setting in Iran. Using a survey-based approach among HCPs in four hospitals in Tehran and using the Copenhagen Burnout Inventory (CBI), we investigate (i) the prevalence of burnout during the pandemic, (ii) how workplace stressors such as excessive workload, lack of job control, and absence of leadership support relate to burnout, and (iii) which demographic or job-related factors predict higher burnout.

The CBI has been widely used to measure burnout in various work settings. Notably, this study is among the first to utilize a Farsi version encompassing all three dimensions—personal, work, and patient-related—of the CBI. Furthermore, the survey includes both medical and non-medical hospital staff from multiple medical units, allowing for a comprehensive analysis of work characteristics, such as providing frontline help to COVID-19 patients, in addition to socio-demographic variables. Given the significant adverse consequences of HCPs’ burnout on health systems, the findings of this study hold substantial importance for healthcare managers and policymakers. Particularly in middle- and low-income economies, where sustaining the core functions of hospitals is already a challenge, improving the effectiveness of health services by promoting a healthy work environment for personnel should be a priority for healthcare policymakers. By identifying key work stressors and proposing tailored organizational interventions, this study offers practical insights for mitigating burnout among HCPs—both in the post-pandemic era and in preparation for future public health emergencies.

## Materials and methods

### Survey design and study population

Iran’s hospital system consists of four main types. State hospitals, which operate under the Ministry of Health and Medical Education, serve as training centers for medical staff. Public non-governmental hospitals, primarily located in urban areas, are managed by the Iranian Social Security Organisation (SSO), the country’s largest health insurer. The SSO provides coverage for all formal employees, except civil servants and service personnel. The remaining hospital types include private hospitals and military hospitals, the latter serving members of the armed forces and their families. This study focuses on two public hospitals managed by the SSO and two private hospitals. The two public hospitals are larger than the two private ones in terms of both bed capacity and staff size. The two public hospitals have a number of 2737 (hospital 1) and 1028 (hospital 2) HCPs respectively, while the private hospitals have 325 (hospital 3) and 263 (hospital 4) HCPs. Participants were recruited from a total population of 4,353 clinical and non-clinical staff across the four hospitals. In the two public hospitals, sampling was conducted in collaboration with IT centers, which maintain access to staff data. A random selection was carried out using the internal IT system with the aim to invite 600 (hospital 1) and 500 (hospital 2) HCPs for participation. In contrast, given that the private hospitals employed fewer than 500 staff members each, all personnel were invited to participate. Out of 4,353 eligible staff across four hospitals, 1,688 were invited (random selection in larger hospitals, all staff in smaller hospitals), of which 723 participated in the survey (43% response).

The recruitment and survey process took place between November and December 2020, during the third wave of COVID-19. Since the respondents were non-native English speakers, we translated the questionnaire into Farsi. To ensure its validity, we followed Brislin’s^25^ five-step procedure: (1) translating the English questionnaire into Farsi; (2) back-translating the questionnaire from Farsi into English by bilingual translators; (3) comparing both versions to ensure semantic accuracy and eliminate potential bias; (4) pilot testing with 17 HCPs to check for comprehensibility and plausibility; and (5) soliciting feedback from the respondents and making minor modifications. Additionally, a panel of experts reviewed the questionnaire for face validity.

### Measurement

The survey utilized the CBI, developed by Kristensen et al. (2005), to assess burnout among HCPs. The CBI comprises 19 questions grouped into three sub-dimensions: a six-item generic scale to measure personal burnout, a seven-item work-related burnout scale, and a six-item patient-related burnout scale. While all questions center on fatigue and exhaustion, the key distinction is the attribution of these conditions to personal, work-related, and patient-related domains^26^. To limit our scope to COVID-19-related burnout, we modified the questionnaire by adding the statement "during the COVID-19 pandemic" to all 19 items.

For each question, we provided five response categories on a 5-point Likert scale, asking about frequency (12 items) or intensity (7 items). The response categories were (1) always/to a very great extent, (2) often/to a great extent, (3) sometimes/somewhat, (4) rarely/to a small extent, and (5) never/almost never/to a very small extent. Responses were scored from 0 to 100, with category 1 scoring 100 and category 5 scoring 0. Scores on the CBI range from 0 to 100, with scores between 50 and 74 indicating moderate burnout, scores between 75 and 99 indicating high burnout, and a score of 100 indicating severe burnout. The CBI is a validated instrument for measuring burnout, with demonstrated reliability and validity in various languages, countries, and healthcare professions.

Our survey incorporated three items drawn from the Job Demand-Control-Support model to assess respondents’ experiences of workplace stressors. These items assessed (1) increased workload; (2) control over job tasks; and (3) communication with leadership (e.g., supervisors). To minimize respondent burden, each stressor was assessed with a single question. We obtained information on whether work hours had significantly increased compared to the reference of either no increase or a somewhat insignificant one. Respondents were also asked whether they feel unable to control their tasks and workload and whether they would inform their supervisor if they experienced burnout. For the latter two questions, we provided response categories on a 5-point Likert scale ((1) never, (2) seldom, (3) sometimes, (4) often, (5) always). We created dummy variables for loss of job control and likelihood of communicating with a supervisor, assigning a value of 1 if response categories (4) or (5) were chosen and 0 otherwise.

Since effective communication is a critical component of leadership^27^, in line with Beehr et al.^28^, we operationalized workplace support through communication with leaders. Additionally, the survey captured several job characteristics, such as whether HCPs were clinical or non-clinical staff members and whether or not they provided frontline assistance to COVID-19 patients (provider vs. non-provider). Furthermore, information on socio-demographic characteristics, such as age, gender, marital status, number of children, highest educational attainment, and years of experience, was incorporated into the survey.

### Survey administration

The survey was administered between November 1 and December 30, 2020, during the third wave of the COVID-19 pandemic in Iran. Before conducting the survey, we consulted hospital authorities to identify the most appropriate and feasible data collection methods. As a result, we implemented different modes of administration across the four hospitals: in one public non-governmental hospital, we distributed the survey by sending an email invitation with a survey link through its internal electronic system; in the other public hospital, we conducted telephone interviews. In the two smaller private hospitals, respondents completed a written survey. Despite these variations in administration, an identical questionnaire was used across all hospitals.

Ethics approval was obtained from the ethics committee of the University of Hohenheim (reference: 2020–0928), and all participants provided informed consent. The survey was conducted in accordance with the ethical principles outlined in the World Medical Association’s Declaration of Helsinki. The data was stored in anonymised format and only the authors had access for the purpose of analysis (secure storage).

### Statistical analysis

We conduct a statistical analysis of the data, beginning with a descriptive analysis (means, proportions, and the relative frequency distributions for the 19 items of the CBI) to characterize the sample and burnout prevalence. To assess internal validity, we calculate Cronbach’s alpha for the sub-dimensions of personal, work-related, and patient-related burnout. In the next step, we present the mean values and 95% confidence intervals for the overall CBI score and its sub-dimensions, stratified by various factors, including staff type (clinical vs. non-clinical), COVID-19 patient contact (frontline healthcare provider vs. non-provider), increase in work hours (none vs. significant), job control (control vs. no control), and ability to communicate with management (less likely vs. likely to speak to a supervisor).

Subsequently, we perform a multivariate linear regression analysis to identify factors contributing to burnout. Using the overall CBI score and the three sub-dimensions as continuous dependent variables, we conduct ordinary least squares (OLS) regressions including staff type, COVID-19 patient contact, workload, job control, ability to communicate, and socio-demographics as independent variables. Socio-demographics include gender, age, marital status, number of children, education, and years of experience. In addition, we include dummy variables for the hospitals to capture hospital specific characteristics and survey mode. All analyses are performed using the statistical software STATA 16. In our regression analysis with CBI being the dependent variable we yield an analysis sample of N = 670 with non-missing data in all covariates. Detailed characteristics of the participants are summarized in Table 1.

**Table 1:** Characteristics of participating HCPs.

	Proportion/mean
Female [% (n)]	62 (415)
Married [% (n)]	79 (529)
Number of children [mean ± SD]	1.09 ± 0.95
Age [years, mean ± SD]	38.80 ± 7.04
Years of experience [years, mean ± SD]	13.53 ± 6.40
Education [% (n)]	
Diploma, associate	26 (176)
Bachelor	57 (379)
Master & PhD	17 (115)
Increased workload [% (n)]	39 (261)
No control over tasks and workload [% (n)]	37 (248)
Communicate with supervisor [% (n)]	35 (235)
Frontlinehelp [% (n)]	58 (389)
Staff group clinical [% (n)]	78 (523)
Ward [% (n)]	
Inpatient general	30 (201)
COVID-19	6 (40)
Emergency	9 (60)
Surgery	24 (161)
Supportive	31 (208)
Hospital [% (n)]	
1	52 (346)
2	27 (179)
3	16 (106)
4	6 (39)

N = 670. Sample is based on regression for CBI

## Results

Of the 670 respondents with non-missing data, 62% (n = 415) were female and 79% (n = 529) were married. On average, participants had 1.1 (SD = 0.95) children and 13.5 (SD = 6.4) years of experience; mean age was 38.8 years (SD = 7.0) (see Table 1). The majority of respondents (78%; n = 523) was clinical staff and more than half (58%; n = 389) provided frontline help with direct contact to COVID-19 patients. Respondents represented a range of disciplines and exposure settings, reflecting diverse professional and demographic characteristics.

**Fig. 1:**
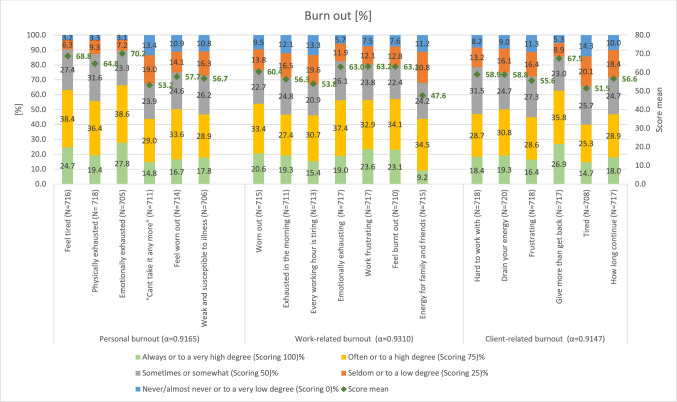
Prevalence of burnout among the surveyed HCPs.

Our descriptive analysis continues by assessing the prevalence of burnout, mapping the relative frequency distribution of response categories for each of the 19 items of the CBI (Fig. 1). Our findings reveal that 67.41% of respondents (452 out 670) scored 50 and above on the CBI, reporting either *sometimes/ somewhat* or *often/to a high degree*, indicating a high level of burnout. For 10 out of the 19 burnout items, at least half of the respondents reported experiencing burnout "always/to a very high degree" or "often/to a high degree", highlighting alarming levels of burnout. Among the remaining nine items, the proportion of respondents using these categories is 40% or more. As shown in the figure (right axis), the mean score across all 19 items exceeds 50, with an overall CBI score of 59.6, indicating moderate to severe symptoms among nearly two-thirds of respondents across all three CBI dimensions. The mean scores for the personal, work-related, and patient-related burnout are 61.8, 58.9, and 58.1; out of 670 respondents 72.86% (n = 488), 69.87% (n = 468), and 65.37% (n = 438) report moderate to high levels of burnout (CBI ≥ 50) in these sub-dimensions, respectively. All sub-dimensions exhibit excellent internal validity, with Cronbach’s alpha values > 0.9 which is slightly higher than in other studies using the CBI questionnaire in Persian language^29–31^.

**Fig. 2:**
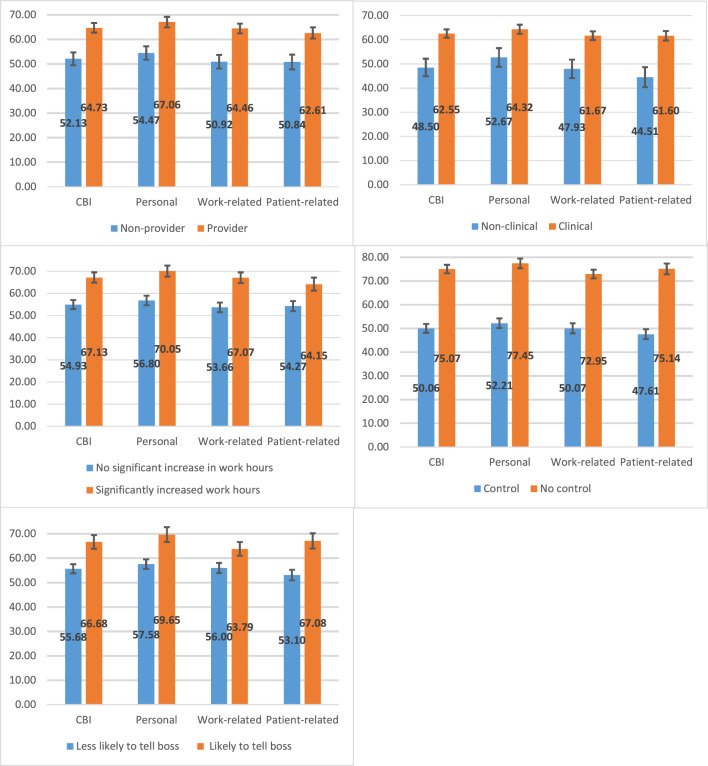
Burnout by staff type and work characteristics (mean scores and 95% CI).

In the subsequent step, we disaggregate the overall mean score of CBI and its sub-dimensions by several organizational stressors and job characteristics. Specifically, we examine (i) whether respondents’ work hours have been significantly increased; (ii) whether respondents have control over their tasks or job; (iii) whether respondents are likely to communicate with their leader/supervisor if they are experiencing burnout symptoms; (iv) staff type (clinical vs. non-clinical); and (v) whether respondents are frontline healthcare providers for COVID-19 patients. Figure 2 presents the stratified mean scores and 95% confidence intervals.

Regarding job characteristics and organizational stressors, we observe similar mean scores for the overall CBI and the sub-dimensions, with substantial differences in the groups we have stratified. We find markedly higher levels of burnout among clinical staff compared to non-clinical staff (CBI mean score: 62.6 vs. 48.5). Similarly, average burnout levels are higher among frontline healthcare providers (CBI mean score: 64.7) than among non-providers (52.1).

HCPs who experienced a significant increase in working hours reported higher average levels of burnout (CBI mean score: 67.1) compared to those who did not (54.9). It is therefore not surprising that the highest burnout scores are observed among HCPs who expressed a lack of control over their tasks with a CBI mean score of 75.1 compared to 50.1 of those who are in control over their tasks. In this group with lack of control over their tasks, the score for the overall CBI, as for the sub-dimensions of personal- and patient-related burnout, exceed 75, corresponding to a high level of burnout. We also note that burnout levels in this group are much higher than in groups with more control over their tasks and workload. Further analysis of whether respondents are likely to communicate their burnout to their supervisor reveals substantially higher levels of burnout among those who are likely to confide in their supervisor (CBI mean score: 66.7 vs. 55.7).

Turning to the results of our regression models, we observe that they confirm the findings of our descriptive analysis within a multivariate linear regression framework that accounts for additional control variables (Table 2).

**Table 2:** Regression analysis.

	CBI		PB		WR		PR	
	Coef	SE	Coef	SE	Coef	SE	Coef	SE
Constant	43.365***	5.269	43.047***	5.743	42.398***	5.898	43.105***	6.159
Female [0 = no; 1 = yes]	5.302***	1.496	5.290***	1.629	6.353***	1.679	4.103**	1.751
Married [0 = no; 1 = yes]	3.505*	1.904	4.869**	2.073	3.734*	2.134	2.308	2.229
Number of children	− 1.512	0.942	− 2.688***	1.026	− 1.797*	1.058	− 0.207	1.105
Age	− 0.190	0.159	− 0.160	0.173	− 0.155	0.178	− 0.239	0.186
Years of experience	0.144	0.173	0.165	0.189	0.086	0.194	0.206	0.203
Education [ref.: diploma, associate)]								
Bachelor	3.924**	1.707	2.660	1.859	3.785**	1.916	5.277***	1.998
Master & PhD	− 2.142	2.217	− 3.397	2.415	− 2.514	2.487	− 0.245	2.595
Staff group clinical [ref.: non-clinical]	5.219***	1.970	3.655*	2.149	4.907**	2.211	7.138***	2.310
Frontlinehelp [0 = no; 1 = yes]	3.333**	1.547	3.603**	1.681	5.230***	1.736	1.145	1.812
Increased work hours [0 = no; 1 = yes]	4.031***	1.524	5.440***	1.658	5.613***	1.710	0.974	1.783
No control over tasks and workload [0 = no; 1 = yes]	18.469***	1.528	17.573***	1.659	17.616***	1.715	20.630***	1.790
Communicate with supervisor [0 = no; 1 = yes]	3.655**	1.529	3.909**	1.666	2.164	1.714	5.546***	1.791
Ward [ref.: inpatient general]								
COVID-19	5.578*	3.196	7.097**	3.485	2.779	3.588	7.607**	3.748
Emergency	4.343*	2.611	5.981**	2.825	2.900	2.931	4.288	3.038
Surgery	− 0.672	1.884	0.951	2.046	− 0.617	2.115	− 2.199	2.206
Supportive	− 1.244	2.055	2.737	2.241	− 0.717	2.307	− 5.760**	2.409
Hospital [Ref.: 1]								
2	− 2.193	2.972	1.006	3.239	− 2.845	3.337	− 4.407	3.486
3	2.010	1.865	6.396***	2.032	− 2.245	2.089	1.883	2.182
4	− 6.179***	2.015	− 6.723***	2.191	− 6.454***	2.253	− 5.466**	2.348
Number of observations	670		674		672		674	
F-stat	24.901		21.649		18.078		22.293	
R^2^	0.421		0.386		0.345		0.393	

*** p < 0.01, ** p < 0.05, * p < 0.1

Consistent with our previous analysis, increased work hours are associated with significant higher levels of burnout (p < 0.01), with patient-related burnout being the only insignificant sub-dimension. The largest and most consistently significant coefficients (p < 0.01) are observed among HCPs who report a lack of control over their tasks and workload, with large coefficients reflecting the high values seen in the descriptive analysis and underscoring the severity of job control loss as a source of burnout.

Interestingly, we also find that HCPs who are inclined to communicate with their supervisor about burnout experience higher levels of burnout. This might suggest that these individuals acknowledge their distress and are more likely to seek support—or alternatively, that the communication may not be adequately supportive. This could indicate a potential gap in effective leadership support.

Additionally, as presented in Table 2, clinical staff exhibit significantly higher levels of burnout than non-clinical staff across all dimensions (p < 0.01), as reflected in the positive and significant coefficients. Similarly, frontline HCPs who provide care for COVID-19 patients experience higher levels of burnout, although the coefficient for patient-related burnout is not significant.

To further explore the association between burnout and demographic factors, our analysis includes several control variables. Controlling for gender, relationship status, education, years of experience, and age, we find significant variations in burnout across demographics. Specifically, female HCPs report higher levels of burnout than their male counterparts (p < 0.05), as indicated by consistently lower coefficients for male respondents across all dimensions. Additionally, married HCPs experience higher levels of burnout than single HCPs, although the coefficient is not significant for patient-related burnout. Interestingly, the number of children was negatively associated with burnout, with significant effects observed for personal and work-related burnout (p < 0.01 and p < 0.1, respectively). Conversely, there was no significant relationship between age and burnout, or between years of experience and burnout. Regarding educational attainment, those with a master’s degree or higher (e.g., MD, Ph.D.) exhibit lower levels of burnout, with negative but insignificant coefficients observed in all dimensions. In contrast, respondents with a bachelor’s degree report higher levels of burnout, with positive coefficients which are significant across all sub-dimensions (p < 0.05) except for personal burnout.

## Discussion

The findings of this study on Iranian HCPs suggest that burnout is a widespread issue (67.41%), aligning with previous studies conducted worldwide^14,17,19,22^. The results confirm that organizational stressors such as increased work hours, not having control over tasks and workload, and potentially non-effective leadership support strongly predict burnout in an Iranian sample. Groups that are particularly at risk include clinical staff and those providing frontline help, i.e. having direct contact with COVID-19 patients.

Given the distressed state of Iran’s healthcare system, the high rate of COVID-19 infections and mortality, and the country’s infrastructural challenges, this finding is particularly alarming. Shortages of diagnostic and treatment facilities, personal protective equipment, standard isolation rooms, and hospital beds are just some of the infrastructural deficiencies that have overwhelmed Iranian HCPs^31^. In fact, the pandemic-driven surge in demand for medical care has unveiled the underinvestment and systemic shortfalls in the Iranian healthcare system. As a result, overburdened HCPs report negative health outcome and increased rates of burnout. It also reveals that while burnout manifests at the individual level, its underlying causes stem from the systemic and organizational factors. This is reflected in a key finding of this study—the significant association between higher levels of burnout and work-related stressors—specifically, workload, job control, and leadership communication—reinforcing their relevance to HCPs’ burnout in the context of the pandemic.

### Job demand

Our analysis shows a significant association between the high prevalence of burnout and increased workload. This finding is consistent with several studies identifying excessive workload as a major contributor to exhaustion and burnout^2,16,32^. Our findings also reflect a large proportion of HCPs reporting emotional and physical exhaustion, which can largely be attributed to the ongoing shortage of HCPs in Iran^33^. This issue is further exacerbated by the disproportionate demand for medical care as a result of the pandemic, which has imposed an extra 100 h of work per month on Iranian HCPs^34^. Rectifying this issue is particularly difficult for Iran, given that the provision of medical staff is directly tied to the country’s gross national product (GNP) per capita^35^. Marred by sanctions and the accompanying economic fallout, Iran’s health system is squeezed by mounting expenses and plummeting income with deep cuts in hospital billings leading to layoffs and temporary employment^34^. To alleviate HCPs’ workload, effective resource allocation is essential. Short-term measures may include setting fair productivity targets, clearly defining job roles, and offering intrinsic rewards and non-monetary incentives. Long-term strategies should focus on redistributing funds and investing in digital infrastructure to streamline the on-site tasks for HCPs. While optimizing digital tools, such as the utilization of information and communication technology (ICT) in healthcare organizations may seem financially burdensome in the short term, research suggests that it improves the quality of patient care at a reduced cost in the long run^36^.

### Job control

Our results demonstrate that HCPs with less job control were more likely to experience burnout during the pandemic. The COVID-19 crisis has imposed unique challenges for HCPs, including the lack of well-defined clinical guidelines, role ambiguity, and skill-task mismatch, all of which contributed to a sense of limited control over the job and restricted decision latitude (decision-making authority)^31,37^. The ability to make independent decisions about patient care without interference from non-medical legislation is crucial in managing the critical situations^38^. In light of this, implementing regulations that promote greater flexibility in setting charges and tasks during public health emergencies seem to be promising. However, this alone may not be enough to mitigate the effects of burnout if HCPs are not prepared to perform new tasks and to navigate the continuously changing practice environment. Thus, tailored training and coaching programs are necessary, with an emphasis on engaging HCPs in establishing new work requirements and structures as well as shared decision-making procedures. Success in this regard may give HCPs a sense of self-sufficiency and autonomy, leading to a better psychological adjustment to the pandemic and alleviating the effects of burnout. Moreover, our research indicates that HCPs with higher levels of education were less likely to experience burnout; this finding highlights the importance of knowledge in coping with unforeseen circumstances. This knowledge could be leveraged in peer support programs, enabling leaders to enhance clinical interactions and promote HCPs’ professional development where their job control is limited.

### Leadership communication

Our findings unexpectedly suggests that HCPs with severe burnout symptoms were more likely to express their concerns to their supervisors. However, a primary occupational concern for HCPs in Iran is the "lack of leadership support and acknowledgement" from hospital administrations and department leaders/ supervisors. This may cause HCPs to feel undervalued and unsupported, with adverse effects on their mental health and the increased likelihood of burnout. The high rate of burnout among surveyed HCPs illuminates the critical role of leadership characteristics, particularly communication skills. Leaders of health organizations, primarily leaders with a medical education, often lack essential managerial skills and knowledge^39^. Therefore, targeted education is necessary to develop, prepare, and equip these leaders for managerial roles. This education should include effective leadership communication, characterized by consistency and mutuality, promoting the perception of being valued, respected, and fairly treated^4^. Clear and compassionate conversations, along with acknowledgement and appreciation for employees expressing distress and resentment, ensure a safe and supportive work environment that is conducive to HCPs connecting with their managers and communicating without fear of dismissive language or retaliation.

Table 3 summarizes the key work stressors contributing to HCPs’ burnout, the underlying drivers of pertinent issues, and recommended interventions. The table outlines intervention strategies at three levels: (i) adjusting workload (redistribution, digital tools, incentives), (ii) enhancing job control (shared decision-making, training), and (iii) improving leadership communication (training for leaders, recognition programs, psychological safety). The proposed interventions have the potential to yield substantial economic benefits by reducing job turnover, improving job satisfaction, and enhancing productivity. However, the financial and productivity gain will depend on the specific interventions implemented, the size of the healthcare organization and other contextual considerations.

**Table 3:** Determinants of burnout at workplace, drivers, and recommended construct for interventions aimed.

Work stressors/organizational contributors	Underlying drivers	Organization—directed interventions
Excessive workload	Personnel shortages Liquidity shortages Disproportionate demand	Appropriate distribution of job roles Promoting flexibility and tailoring work Establishing fair productivity targets Optimizing digital tools/ infrastructure that allows remote work
Limited job control	Limited sense of autonomy and discretion (or sense of displacement) Skill-task mismatch Role ambiguity Lack of peer support	Engagement in establishing work requirements and structure Shared decision making Peer support programs Trainings and coaching programs focused on reducing role ambiguity
Lack of leadership support	Toxic work environment Not feeling heard or seen Dismissive language Lack of integrity	Training leaders on critical leadership knowledge and skills Increasing leadership acknowledgement and appreciation Creating psychological safety via conversations Fostering work relationships appropriately among the team Offering rewards and incentives

Our study further identifies factors associated with burnout in HCPs. Specifically, the clinical specialties with the highest rates of burnout among HCPs are those involving frontline services in COVID-assigned wards and emergency medicine. This pattern mirrors the results of other studies, highlighting similarly high burnout rates among frontline providers, especially those engaged in emergency medicine and COVID-19 treatment settings^16,22^. This may be due, in part, to fear of workplace COVID-19 exposure and family transmission, as well as social stigma and discrimination against frontline HCPs. While the former is a source of anxiety that aggravates concerns among HCPs themselves^40^, the latter reflects public’s fear of HCPs as potential sources of COVID-19 infection. Such stigma and harassment can force frontline HCPs, particularly females, into social isolation that undermines their mental health^41^. To address such social stigma, raising public awareness through the media and providing the public with appropriate health education have proven effective^42,43^. In any case, these results give us valuable insights into which specialties are at higher risk of burnout and which groups should be focused on when considering possible interventions.

It is therefore worth emphasizing the detrimental impact of the pandemic on female HCPs; in addition to the extended caregiving and household responsibilities resulting from the surge in COVID-19 patients, gender-related behavioral patterns may also contribute to this disparity. Female HCPs often adopt a more patient-centered and empathetic approach, which has been shown to result in greater patient engagement^44^. As a result, the emotional burden of high mortality rates among COVID-19 patients and the need to prioritize care may place female HCPs at a greater risk of burnout. Therefore, the implementation of gender-specific interventions is crucial^45,46^.

The results of this study elucidate the critical context of the challenges experienced by HCPs from the resourced-constrained context of Iran during the COVID-19 pandemic, shedding light on how key work stressors contribute to the increased risk of burnout. The theoretical implications of this study for occupational health and burnout research are worth noting. First, given the strong association between HCPs’ burnout and organizational factors found in this study, our findings build on the existing literature by supporting the relevance of the workplace stressors to burnout, particularly in the healthcare sector. Second, while factors associated with burnout has been studied in numerous settings in developed economies and within non-pandemic contexts, this research contributes to our understanding of the impact of occupational stressors in low- and middle-income economies, particularly in the unique crisis setting of the COVID-19 pandemic. From a practical perspective, the discovery that workplace stressors play a central part in HCPs’ burnout should be communicated to leaders of health care institutions and policy makers. This evidence-based framework provides the essential knowledge they need to make informed decisions for setting priorities that will address the burnout epidemic in HCPs at scale, and to ensure preparedness for future public health emergencies. This would benefit not only individual HCPs but also the economy and public health.

### Limitations and further research directions

While our study provides valuable insights into the factors contributing to burnout among HCPs, a few limitations should be acknowledged. First, due to the cross-sectional design of our study, causation could not be established. Regarding representativeness of the sample, our population indicates a 2.7 ratio of hospital staff (clinical and non-clinical) to beds, consistent with Iran’s national hospital indicator ratio of 2.57^47^. Therefore, we believe that our dataset enables a sufficiently robust analysis to make meaningful comparisons. However, our findings are based on surveys conducted in four hospitals in Tehran during the third peak of the COVID-19 pandemic, which could limit the generalisability to other regions and times.

Furthermore, due to the heavy workload and time constraints faced by HCPs during the third peak of the pandemic at the time of data collection, we assessed work stressors using single-item measures to save respondents’ time. Although the efficiency of single-item measures in assessing various constructs is widely recognized^48,49^, using multiple-item measures is generally preferred. Thus, the results could potentially be subject to residual confounding due to imperfectly or unmeasured variables. Another potential bias in the results could arise from self-selection (voluntary participation)—for example, HCPs suffering from burnout may have been more or less likely to participate in the study. Finally, while an identical questionnaire was used for all participants, mode effects are another potential limitation although we control for the hospitals and therewith for mode of interview.

Despite these limitations, the widespread prevalence of burnout among HCPs highlights the need for data on the impact of interventions and the challenges arising during the implementation. Further longitudinal studies could build upon the findings of this study, exploring the impact of the recommended interventions on HCPs’ burnout and the health institution’s outcome, with particular attention to contextual and institutional differences.

## Conclusion

Rooted in the occupational context and being more a function of the work environment than the individual, burnout among HCPs poses a significant challenge to the advancement of health systems. As such, it merits extra attention from health care policy makers and organizational leaders. Our study highlights that organizational resources such as managerial support, job control, and workload management are strongly associated with the prevalent burnout among HCPs, with higher rates observed in females and frontline staff. The present study proposes that addressing burnout requires organization-focused interventions aimed at supporting HCPs in their recovery and reintegration post-pandemic. These insights suggest that even beyond COVID-19, healthcare systems should proactively address workload, autonomy, and communication to build resilience in any future health emergency. In terms of preparedness for any future emergency, additional personnel capacity, material resources and organisational contingency plans should be provided and made available for immediate use when needed.In this context, organization-focused interventions and the role of immediate supervisors and leaders as agents of change become paramount. They are a valuable resource in policy implementation, and their characteristics and professional pursuits can promote a healthy work environment that not only improves HCPs’ well-being, but also yields broader benefits for the economy and public health.

## Data Availability

The data sets generated and analyzed during the current study are available from the corresponding author on reasonable request.
